# Unilateral Dorsal Root Ganglion Stimulation Lead Placement With Resolution of Bilateral Lower Extremity Symptoms in Diabetic Peripheral Neuropathy

**DOI:** 10.7759/cureus.10735

**Published:** 2020-09-30

**Authors:** Kenneth B Chapman, Bert-Kristian W Van Roosendaal, Noud Van Helmond, Tariq A Yousef

**Affiliations:** 1 Pain Management, Hofstra Medical School/Northwell Health Systems, New York, USA; 2 Anesthesiology/Pain Management, New York University Langone Medical Center, New York, USA; 3 Anesthesiology, Radboud University Medical Center, Nijmegen, NLD; 4 Anesthesiology, Cooper Medical School of Rowan University, Camden, USA; 5 Pain Management, The Spine and Pain Institute of New York, New York, USA

**Keywords:** dorsal root ganglion stimulation, painful neuropathy, dorsal root ganglion, diabetic peripheral neuropathy, sympathetic nervous system, dpn, drg-s, drg, pain, pain management

## Abstract

Dorsal root ganglion stimulation (DRG-S) is a form of neuromodulation that can target specific dermatomes to obtain better coverage of the distal extremity. Previously proposed mechanisms of action for DRG-S focused on the dorsal root ganglion (DRG) itself, without consideration of orthodromic effects in the dorsal horn and antidromic effects on the nerve root and sympathetic chain. Diabetic peripheral neuropathy (DPN) is an axonal neuropathy that affects around half of all patients with diabetes mellitus, causing severe pain and sensory impairment in the distal extremities. We present a case of a patient with DPN in both feet, in addition to low back pain, who underwent a DRG-S trial with right T12 and S1 leads. The trial was performed unilaterally for seven days, allowing the patient to compare the treated versus the untreated (left) side. Pain, disability, general health status, and quality of life measures improved significantly. In addition to the significant pain relief in the low back and feet, the patient had near resolution of other DPN-related symptoms, including numbness, bluish discoloration, and allodynia of both feet. He also demonstrated functional and psychological benefits with only a single-sided lead. Overall, the placement of unilateral T12 and S1 DRG-S leads resulted in symmetric improvement of DPN symptoms. A possible mechanism of action is antidromic propagation of action potential signaling into the sympathetic chain to a central ganglion and then to the contralateral sympathetic chain. Given the DRG's ability to directly affect afferent sympathetic fibers with low-frequency stimulation, DRG-S may be an effective neuromodulatory treatment for DPN.

## Introduction

Dorsal root ganglion stimulation (DRG-S) is an effective form of neuromodulation therapy for several refractory chronic pain syndromes [1-4]. Compared to dorsal column spinal cord stimulation, DRG-S may provide better relief for neuropathic pain syndromes of the distal extremities due to the ability to specifically target nociceptive transmission in DRGs associated with the dermatomes of the feet [5]. Moreover, DRG-S likely modulates neural transmission beyond the previously proposed local mechanisms at the dorsal root ganglion (DRG) itself [6] through orthodromic effects at the dorsal horn and antidromic effects in the nerve root and sympathetic chain.

Diabetic peripheral neuropathy (DPN) is a length-dependent axonal polyneuropathy that begins in the distal lower extremities. An estimated 50% of patients develop DPN within 25 years after the initial diagnosis of diabetes mellitus. Of these, up to 40-50% will develop severe refractory pain [7]. Traditional spinal cord stimulation (SCS) has been used to treat DPN with some benefit, with a systematic review demonstrating >50% pain relief in 63% of patients [8].

This case study reports on the near-resolution of bilateral DPN symptoms from unilateral DRG-S trial at the T12 and S1 levels and reviews possible mechanisms responsible for this phenomenon. The patient described in this report provided written informed consent for the publication of this report.

## Case presentation

A 61-year-old diabetic male patient presented with chronic low back pain and DPN. Significant comorbidities included depression and anxiety. He rated his pain 8/10 cm on visual analog scale (VAS) for the low back and 9/10 cm in the feet. He described his pain in the feet as constant, burning, and throbbing, with intermittent shooting pain between the toes in both feet. Additionally, he complained of numbness and tingling, blue discoloration, and a “cold feeling” in his feet in a symmetrical stocking distribution. His low back pain was non-radiating and characterized as aching and throbbing. He had foot pain at rest and could not walk more than 50 feet without stopping. Physical examination revealed dusky and blue feet that were cold to touch, with reduced sensation to pinprick and vibration, allodynia, and paresthesias present symmetrically in both feet. His lumbar spine showed a decreased range of motion with pain, positive facet loading maneuvers, and tenderness to palpation of the lumbar paraspinal muscles. An MRI of the lumbar spine demonstrated facet arthropathy with foraminal stenosis.

**Figure 1:**
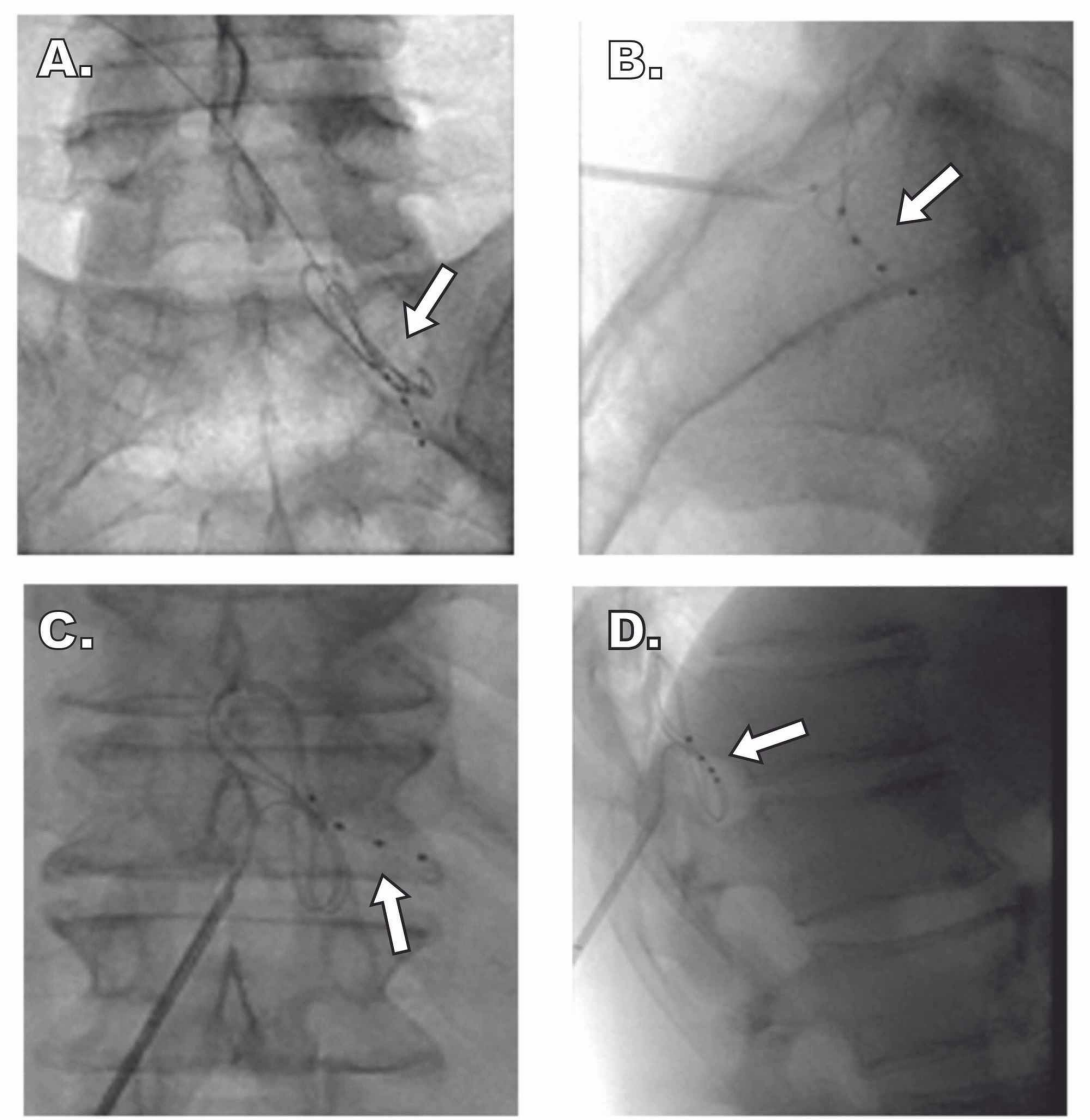
Fluoroscopic Imaging of DRG Lead Placement Unilateral lead location at the right T12 and S1 DRG in a patient who experienced almost complete resolution of bilateral foot pain. Arrows indicate placement of the electrodes in proximity to the DRG within the intervertebral foramen. (A and B) AP and lateral views of S1 DRG lead placement, respectively. (C and D) AP and lateral views of T12 DRG lead placement, respectively. DRG, dorsal root ganglia; AP, anteroposterior

The patient failed multiple conservative treatments for his bilateral foot pain, including gabapentinoid and non-steroidal anti-inflammatory medications. He experienced back pain relief from lumbar medial branch blocks and radiofrequency ablations. However, the corticosteroids would consistently elevate his blood glucose concentrations for several days after these procedures. Considering we were unable to effectively treat his foot pain, combined with the blood glucose elevation from the corticosteroids used for his back pain treatment, we explored other treatment options to address both problems effectively. We offered the patient a trial of DRG-S, given our positive experience with DRG-S for both neuropathic pain and axial low back pain, after he received psychological evaluation clearance for his baseline depression and anxiety. As per usual practice at our institute, regardless of bilateral symptoms, we performed a unilateral stimulation trial for seven days. A unilateral trial has several advantages over a bilateral one, including the ability for the patient to compare the treated side with the untreated side and a decreased risk of complications and post-procedural pain. Leads were trialed at the right T12 and S1 to cover the low back and distal extremity pain (Figure 1). During the single-sided trial, DRG-S settings included a frequency of 20 Hz, a pulse width of 260 us, and an amplitude of 0.425 mA.

We collected VAS, EuroQol-5D (EQ-5D), 36-Item Short Form Survey (SF-36), and Oswestry Disability Index (ODI) data at baseline and at the end of the seven-day trial as part of standard of care (Table 1).

**Table 1:** VAS, EQ-5, SF-36, and ODI data Pain (VAS), back pain related disability (ODI), global health status (EQ-5D), and physical and mental functioning (SF-36) at baseline and completion of the dorsal root ganglion stimulation trial DRG-S, dorsal root ganglia stimulation; VAS, visual analog scale; ODI, Oswestry Disability Index; EQ-5, EuroQol-5D; SF-36, 36-Item Short Form Survey

Measurement	Baseline	End of DRG-S trial
Pain, VAS (in cm)		
Back	8	1
Feet	9	0
Back pain related disability, ODI	72	16
Global health status, EQ-5D	0.15	0.88
Quality of life, SF-36		
Physical component score	16	50
Mental component score	24	63

During the trial, DRG-S lead placement at the right T12 and S1 resulted in a substantial decrease in pain in both feet and in a decrease in low back pain. Improvements in back pain, related disability, and quality of life accompanied the improvements in pain. We observed an improvement in skin color in both feet, and the allodynia and numbness in both feet were resolved. Furthermore, the patient could walk without limitations and participate in other physical activities that he was previously restricted from doing.

## Discussion

We observed a profound bilateral treatment effect with unilateral T12 and S1 DRG-S in a patient with DPN. An extensive literature search found only one other published case report describing bilateral pain relief with unilateral DRG lead stimulation involving a patient with bilateral inguinal neuropathy [9].

Diabetic neuropathy is characterized by autonomic dysfunction and microangiopathic disease [10]. Chemical and thermal neurolysis of the lumbar sympathetic chain have yielded positive results in patients with DPN [11]. As the sympathetic nervous system mediates vascular tone, modulation of the sympathetic nervous system with DRG-S may induce vasodilation. Those include filtering high-frequency afferent signals at the dorsal root ganglia [12], antidromic propagation of low-frequency action potentials, inhibitory signals into the sympathetic chain, and dorsal root reflex, which is a direct activation of afferent sympathetic fibers that have the ability to act as efferent fibers and release transmitters [13].

**Figure 2:**
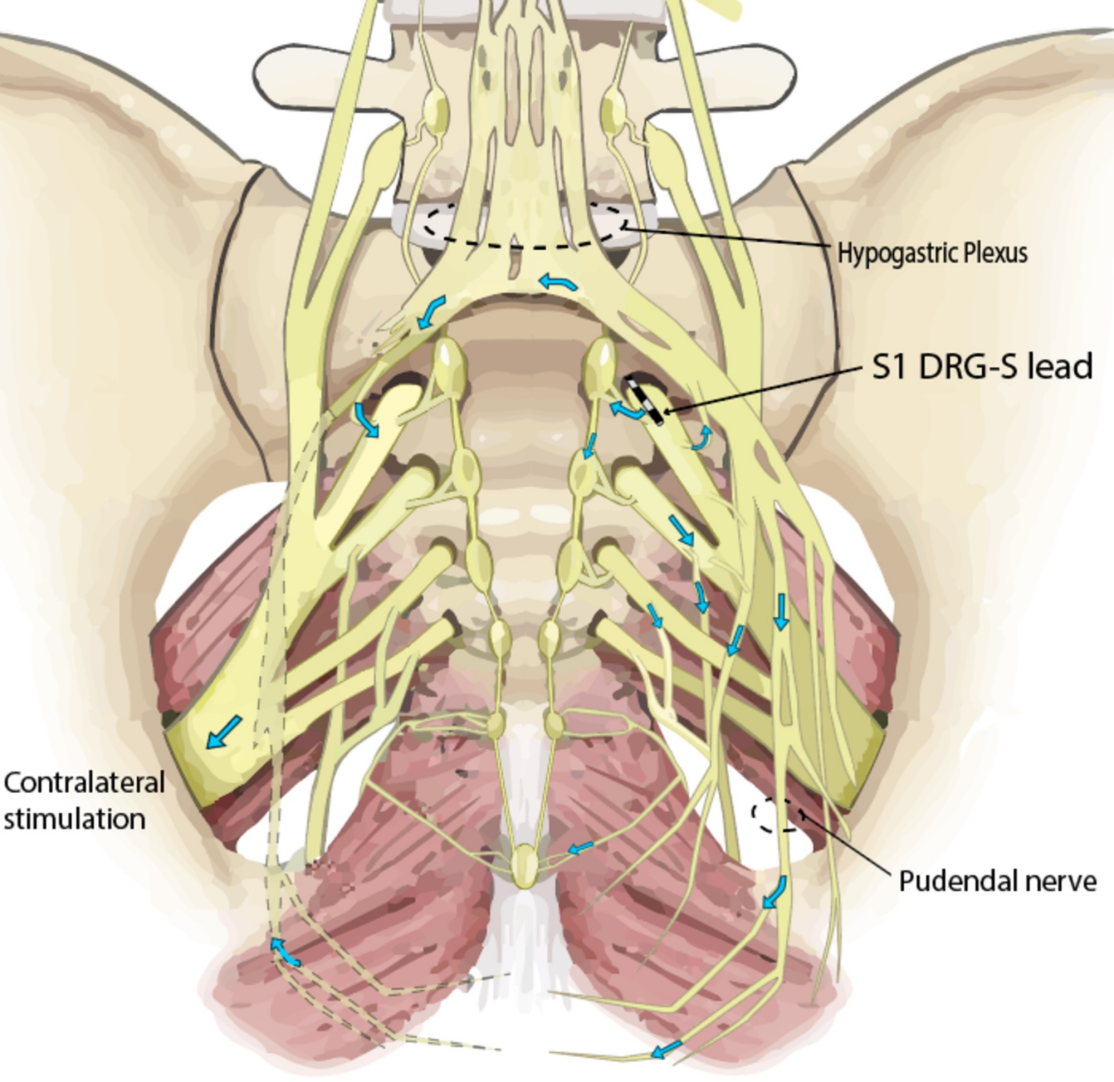
S1 Dorsal Root Ganglion Stimulation S1 dorsal root ganglion stimulation lead in place demonstrating antegrade propagation of action potentials and crossover to the contralateral side through the sympathetic nervous system (figure adapted with permission) [14].

We surmise the mechanism of action underlying the observed bilateral changes in the patient’s feet to be sympathetic modulation at the DRG with crossover fibers in the sympathetic chain and/or the sacral plexuses. These mechanisms can occur through orthodromic and antidromic effects of electrical stimulation at the DRG, such as those listed above (Figure 2) [14]. Sympathetic crossover has been described in several case reports involving sympathetic nerve blockade with local anesthetics and several rodent studies [15-17], but no cases involving neuromodulation have been reported. Anatomical dissections have also demonstrated sympathetic communication from the contralateral chain [18]; however, microscopic interactions between neuronal interfaces in the central ganglions, namely celiac, hypogastric, and ganglion impar, also require consideration.

In addition to the sympathetic nervous system collaterals, a central mechanism cannot be excluded as having played a role in the observed treatment effects. Interconnections between the limbic system, hypothalamus, and brain stem structures comprise the central autonomic network, which processes visceral and somatic afferent signals and, in part, controls sympathetic outflow through fibers descending in the dorsal longitudinal fasciculus. Tracer studies indicate that these descending fibers ultimately project bilaterally, albeit with an ipsilateral dominance, onto preganglionic sympathetic neurons located in the intermediolateral nucleus of the spinal cord [19]. Therefore, stimulation of autonomic afferent fibers at a unilateral DRG site may partially modulate bilateral sympathetic outflow through supraspinal circuits with bilateral descending projections, though most effects would occur ipsilaterally.

Beyond the DPN improvements, the patient’s back pain improved significantly during the DRG-S trial, albeit unilaterally. We previously published a case series demonstrating T12 lead placement efficacy in treating chronic axial back pain [2]. The fact that the therapeutic improvement with DPN symptoms was observed bilaterally, while the somatic back pain only improved ipsilateral to lead placement, highlights the different means by which DRG-S exerts its effects. Back pain relief occurs through unilateral DRG and superficial dorsal horn modulation, whereas DPN improvement likely occurs through bilateral modulation of the sympathetic nervous system, as described above.

This case report underscores the role of the sympathetic nervous system in DPN. Given the DRG's ability to modulate afferent sympathetic fibers with very low-frequency stimulation, DRG-S has the potential to be a powerful neuromodulatory tool for DPN. In our experience, an S1 lead is usually sufficient to cover neuropathic pain of the entire foot, including the L4 and L5 dermatomes, as was seen in this case [20].

## Conclusions

We successfully treated bilateral DPN symptoms with a unilateral DRG-S trial. Improvements in functioning accompanied a considerable reduction in bilateral foot pain. Furthermore, ameliorations in skin color, allodynia, and numbness were observed on both feet. More extensive prospective studies exploring the antidromic effects of DRG-S across the sympathetic chain could further elucidate the hypothesized potential mechanisms of action behind DRG-S treating DPN. Furthermore, larger prospective studies exploring the efficacy of DRG-S as a tool to treat diabetic polyneuropathy may support a potential new role for DRG-S.
